# Editorial: Microflora and bacterial translocation in intestinal obstruction

**DOI:** 10.3389/fsurg.2025.1760588

**Published:** 2026-01-08

**Authors:** Gabriel Sandblom, Leila Meiramovna Koishibayeva, Tomas Poskus, Zhandos Muratovich Koishibayev

**Affiliations:** 1 Department of Clinical Science and Education Södersjukhuset, Karolinska Institutet, Stockholm, Sweden; 2 Department of Surgery, Södersjukhuset, Stockholm, Sweden; 3 Astana Medical University, Scientific and Educational Center of Surgery named after G.V. Tsoi, Astana, Kazakhstan; 4 Vilnius University, Vilnius, Lithuania

**Keywords:** dysbiosis, dysmotility, fecal microbiota transplant (FMT), intestinal obstruction, lipopolysaccharide, probiotic, short-chain fatty acids (SCFA)

What happens in the gut does not stay in the gut! The fact that the intestinal microflora is not safely secluded from the rest of the body by the intestinal barrier has gained increasing attention during recent decades. Several reports have focused on the interaction between intestinal function, gut microbiota, and neurologic, immunologic, and cardiovascular host responses. The interaction between the microflora and intestinal motility plays a major role in gastrointestinal health (1, 2). Peristalsis of the digestive tract is essential for effective digestion, absorption of nutrients, and waste elimination, impacting the microbiota (3).

The interaction between intestinal motility and the microbiota is also bidirectional, as the microbiota affects intestinal metabolism and the maturation of intestinal motility. Motility disorders can, in turn, lead to various conditions, such as chronic constipation, irritable bowel syndrome, and chronic idiopathic pseudo-obstruction (4).

Dysbiosis, a disequilibrium between beneficial and harmful microbial populations, can occur for various reasons. The intestinal microbiota may be affected by acute as well as chronic intestinal obstruction, with potentially serious consequences for the host. Etiologies of dysbiosis include inflammatory bowel disease, non-alcoholic fatty liver disease (NAFLD), and intestinal obstruction (Zhang et al.). Dysbiosis is often part of the pathogenesis of sepsis, a potentially lethal condition. In a systematic review, it was found that the number of publications on microbiota and sepsis has increased rapidly since 2011 (Zhang et al.). In the case of acute intestinal obstruction, the intestinal contents cause bacterial overgrowth that eventually may lead to mucosal injury and ischemia, which increases intestinal permeability. In the presence of dysbiosis and loss of the intestinal barrier function, there is an increased risk of gram-negative sepsis.

Several reports have focused on lipopolysaccharide (LPS) as a target for reducing the effects of sepsis originating from the intestines. LPS is a large glycolipid molecule that forms part of the outer membrane in gram-negative bacteria. Perivascular cells and epithelial cells contain tissue factors that create barriers protecting the visceral organs. The physiological responses caused by these tissue factors are usually beneficial for local and early bacterial infection control. However, if the response to LPS is not appropriately regulated, it can cause excessive inflammation and disruptions in microcirculation, leading to a systemic inflammatory reaction with severe consequences. Several therapeutic strategies have focused on the role of LPS rather than microbial control.

Short-chain fatty acids (SCFAs) are among the most common microbial metabolites in the intestines (Zhang et al.). SCFAs interact with the host's immune system and have stimulating as well as inhibitory effects on inflammatory processes mediated by cytokines. Furthermore, the microbiota-gut-brain axis is regulated by circulating SCFAs. They have been suggested as targets for new therapeutic approaches to reduce the effects of sepsis. The role of LPS and SCFA in the pathophysiology of intestinal obstruction, however, remains incompletely understood and warrants further elucidation.

Fecal microbiota transplant (FMT), transferring feces from healthy donors to the intestines of patients is an established treatment for treating overgrowth of Clostridioides difficile. FMT has also been suggested as a means of controlling sepsis (Zhang et al.), based on the hypothesis that dysbiosis plays a crucial role in the pathogenesis bacterial translocation. In patients with systemic immune reactions, the commensal microbiota is rapidly lost, leading to the overgrowth of potentially pathogenic and pro-inflammatory bacteria. Restoring a healthy microbiota through FMT may, in theory, be a straightforward and effective way of reducing the impact of sepsis as it affects the circulating SCFAs and expression of Interferon Regulatory Factor 3. This has, however, not yet been proven to be effective.

In contrast to acute intestinal obstruction, chronic intestinal obstruction causes a more slowly progressing dysbiosis. The chronic changes in gut microbiota, in turn, affect bile acid metabolism and the turnover of short-chain fatty acids, which affects intestinal motility (5). The dysbiosis in chronic intestinal obstruction may also cause malabsorption, nutrient deficiencies (6), and intestinal pseudo-obstruction (4).

Probiotics, intestinal microorganisms with beneficial effects on the host, have been suggested as a treatment for chronic dysbiosis. Probiotics have several crucial functions, including immune regulation, pathogen prevention, and improvement of intestinal barrier function (Zhang et al.). As these functions could reduce the effects of sepsis, they have also been suggested as a therapeutic measure in acute situations. Studies on next-generation probiotic strains are underway to explore various combinations of probiotics to gain a deeper understanding of how they impact sepsis.

The liver and intestines serve as a barrier that restricts the passage of microorganisms and toxins to the systemic circulation but permits nutrients to pass through. This gut-liver axis is crucial for preventing harmful microbes from spreading in the circulation. In sepsis, the gut-liver axis may be disturbed, triggering a pro-inflammatory cascade that may also cause liver inflammation. Liver dysfunction with impaired bacterial clearance and metabolic disturbance further deteriorates intestinal and liver function, leading to coagulation dysfunction, endocrine disorders, metabolic disorders, and large amounts of LPS entering the systemic circulation. New therapeutic targets focusing on the gut-liver axis during sepsis are, however, under development (Zhang et al.)

Future studies may further elucidate the role of gut microbiota in the cardiovascular, immune, endocrine, and central nervous systems. Whether this will also open up targeted treatment remains uncertain, but the interaction between the microbiota and the host seems fundamental to several organ systems.
